# Research progress and application status of organoid in breast cancer subtypes

**DOI:** 10.17305/bb.2024.11450

**Published:** 2024-12-20

**Authors:** Qiuxia Zhang, Min Wang, Li You, Chen Chen, Jia Feng, Miao Song, Kui Yang, Xuexue Liu, Guangrong Li, Jinbo Liu

**Affiliations:** 1 Department of Laboratory Medicine, The Affiliated Hospital of Southwest Medical University, Sichuan Province Engineering Technology Research Center of Molecular Diagnosis of Clinical Diseases, Molecular Diagnosis of Clinical Diseases Key Laboratory of Luzhou, Sichuan, China

**Keywords:** Organoid, breast cancer, BC, molecular subtypes, resistance mechanisms, therapeutic targets, personalized treatment

## Abstract

Breast cancer (BC) is a prevalent malignant tumor that poses a significant health risk to women. The complexity of basic BC research and clinical treatment is influenced by multiple factors, including age, fertility, hormone metabolism, molecular subtypes, and tumor grading and staging. Traditional *in vitro* models often fall short of meeting modern research demands, whereas organoids—an emerging 3D primary culture technology—offer a unique platform that better replicates the tumor microenvironment (TME). Coupled with advances in high-throughput sequencing technologies, organoids have become increasingly valuable in biological and chemical research. Currently, the most widely used organoid model in BC research is the patient-derived organoid (PDO) model, which is generated directly from original tumor tissues. This paper aims to summarize the current status of PDO models across various BC subtypes, highlighting recent advances in genetics, mechanisms of drug resistance, identification of new therapeutic targets, and approaches to personalized treatment. In conclusion, the development of clinical precision medicine urgently requires *in vitro* models capable of accurately simulating the unique molecular subtypes of patients. This review will examine the challenges and future prospects of organoid models in BC research, offering new insights into the fundamental mechanisms of BC and paving the way for more effective personalized therapies.

## Introduction

Breast cancer (BC) is the predominant malignant neoplasm affecting women on a global scale. According to the 2022 global cancer statistics, the incidence rate of female BC (11.5%) has become the most common cancer besides lung cancer. As the most common cancer among women its morbidity and mortality rates continue to escalate annually, underscoring the pressing need to address this pervasive health threat [1]. Despite advances in the field of oncology, the inherent heterogeneity of tumors and their susceptibility to drug resistance pose serious challenges to oncology researchers. The same is true for BC, which requires the development of personalized treatment regimens, and to address this challenge, research in recent years has utilized organoids of tissue origin, fulfilling its potential in both basic and translational research. Organoids have become invaluable tools for studying the mechanisms of BC development and progression, and for drug screening for different subtypes of BC. These applications mark significant progress in unraveling the mechanisms underlying BC and advancing personalized treatment strategies [2, 3]. The purpose of this article is to critically assess the unique advantages of organoids over other *in vitro* models for various subtypes of BC. New insights are provided to explore the pathogenesis of BC, identify new therapeutic targets and develop personalized treatment strategies.

### BC organoids and traditional models

In recent years, the primary models for BC research have included two-dimensional (2D) cellular models, animal models, and patient-derived organoid (PDO) models. While 2D cell culture lines and patient-derived tumor xenografts (PDX) have been effective in advancing BC research, these models have notable limitations. For instance, 2D cell models are constrained by their simplified physiological performance and lack of a tumor microenvironment (TME), which diminishes their clinical relevance and hampers model expansion. Similarly, PDX models, often developed using mouse models, can closely replicate parental tumor characteristics but face challenges due to physiological and microenvironmental differences between human and mouse mammary glands. These differences hinder the accurate reproduction of molecular and cellular mechanisms underlying BC. Additionally, the influence of the host’s immune system on treatment response is a critical factor that cannot be overlooked. PDX models also suffer from low success rates due to individual variability and high transplantation costs, making them unsuitable for establishing comprehensive biological libraries or for high-throughput drug screening [4, 5]. PDOs offer a promising alternative. Organoids are defined as *in vitro* cultured tissue analogs with specific spatial structures formed through the three-dimensional culture of adult or pluripotent stem cells. PDOs faithfully replicate key features of human BC, including histomorphology, immunohistochemistry, and genomics, while also preserving much of the original TME [6]. These attributes confer high clinical relevance, greater modeling success rates, and enable the straightforward establishment of organoid biobanks. Moreover, PDOs hold significant promise for high-throughput drug screening and facilitate research on BC progression, metastasis, drug resistance mechanisms, and personalized treatment strategies. As organoid technology continues to evolve, it is driving advancements in basic research, drug development, and precision medicine for BC [7, 8].

It is important to note that organoids have inherent limitations. Currently, no model perfectly replicates the complexity of *in vivo* tissue. Consequently, results should be interpreted with caution, and *in vivo* validation should be pursued whenever feasible. Nevertheless, organoid technology is still evolving and requires further refinement of bionic features, including the vascular system, multi-organ systems, and the extracellular matrix. Considering these factors, combining gene-editing technology and microfluidic microarray technology with organoid culture may offer a novel approach [9].

### Construction of BC organoids

**Figure 1.**
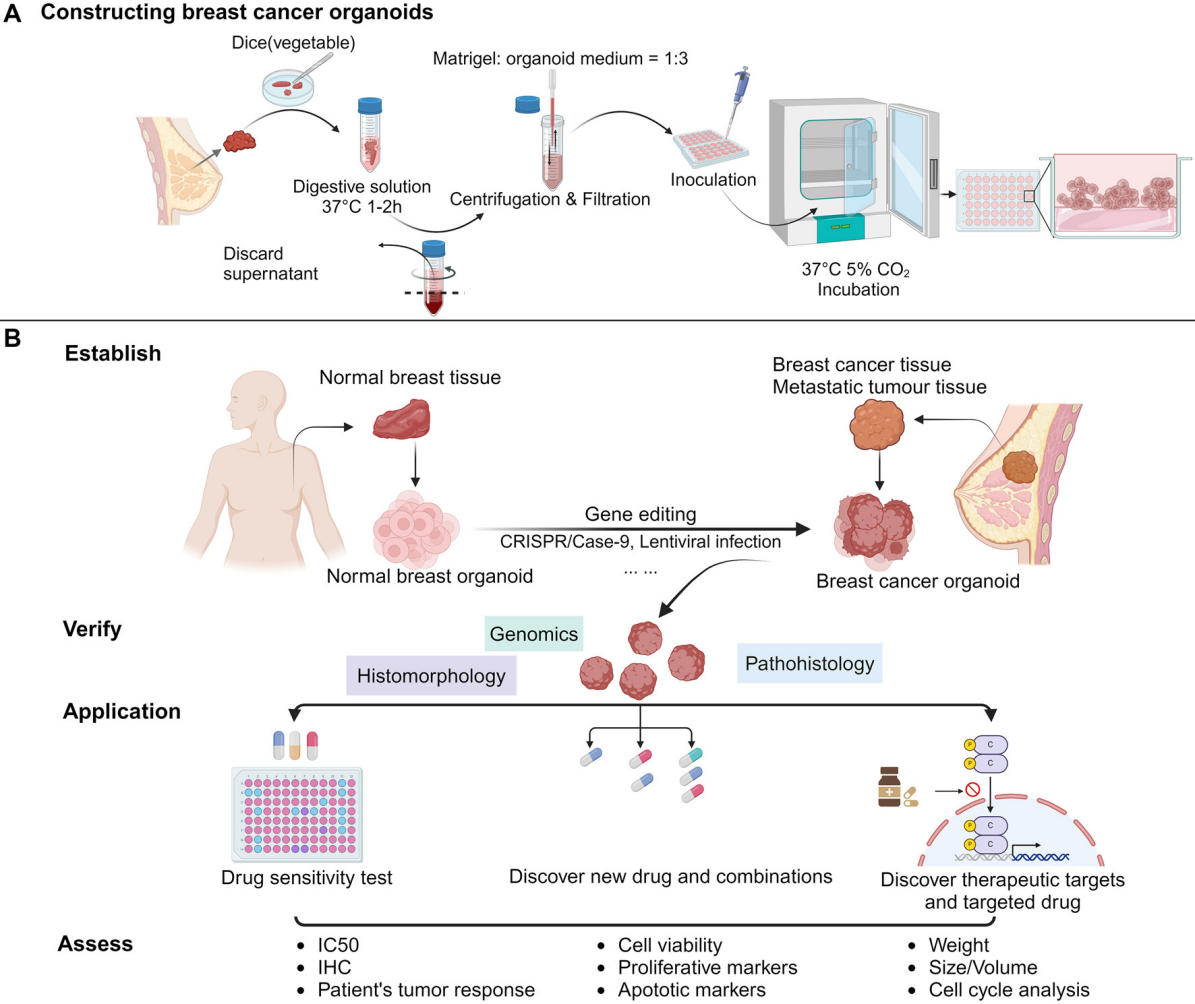
**Overview of BC organoids in terms of establishment, application to drugs, and treatment.** (A) The process of constructing breast cancer patient-derived organoids; (B) After the establishment of organoids, the authenticity of the original tissue of the organoids is often verified through histomorphology, immunohistochemistry, genomics, etc. On this basis, they are applied to drug sensitivity screening to guide patients’ treatment response, discover new drug combinations and compounds, study drug resistance mechanisms explore new treatment targets, etc. Finally, IC50, IHC cell activity, and other outcome indicators are used to evaluate the effectiveness of organoids in research. BC: Breast cancer.

In recent years, a prevailing trend in cultivating tumor PDOs has been the direct culture of tumor tissue, supplemented with cytokines and extracellular matrix components [10–12]. Researchers typically obtain intraoperative tissue samples from BC patients. The primary sources include primary tumor tissue, metastatic tumor tissue, malignant pleural fluid, ascites, and tumor stem cells derived from normal breast tissue [13–17]. These samples undergo a series of processes, including dicing, digestion, filtration, and centrifugation, to isolate a sufficient quantity of stem cells. The resulting cell pellets are resuspended in growth factor-enriched Matrigel (R&D Systems, USA) at a 1:3 dilution with organoid medium. This suspension is then inoculated into 48-well plates and incubated at 37 ^∘^C for 60 min to solidify the Matrigel. Afterward, 300 µL of organoid medium is added to each well, with the medium replaced every three days. Oxygen concentrations and medium change frequency can be adjusted to optimize organoid culture conditions. Organoid construction is considered successful when single cells or small cell clusters grow into multicellular, spherical structures [18, 19]. Organoid media typically consist of a DMEM/F12 basal medium, supplemented with a variety of growth factors, hormones, and inhibitors. These include R-spondin-1, FGF7, neuregulin 1, EGF, FGF10, noggin, A83-01, Y-27,632, SB202190, N-acetyl-L-cysteine (NAC), nicotinamide (NAM), Primocin, GlutaMax, HEPES, and B27. The medium can also be tailored for research purposes through the addition of inhibitory factors, drugs, gene editing technologies, or other modifications. Notably, most researchers have observed that the luminal subtype of BC PDOs grows slowly *in vitro* but can be improved with the addition of NAC and basic fibroblast growth factor. In one study, Tuveson et al. enhanced luminal BC PDOs containing P53 mutations by introducing Nutlin-3a into the culture medium, which promoted P53 expression. Similarly, Johanna F. Dekkers constructed luminal-type BC organoids by transfecting tumor suppressor (TS) genes associated with BC (e.g., TP53, PTEN, RB1, and NF1) to investigate their genetic drivers further. This body of evidence highlights the potential for combining normal BC organoids with gene-editing technologies to explore the genetic factors driving early BC development, a critical avenue for advancing BC gene therapy [12, 20–22]. In contrast, other BC PDO subtypes tend to be more successfully constructed using the aforementioned protocols (Figure 1A). Despite the impressive success rate of up to 80% for constructing BC organoids, challenges persist. The steps and difficulties involved in organoid culture remain consistent, regardless of the molecular subtype or grade of the original BC tissue [21].

Research by Campaner et al. [23] has highlighted that the aggressiveness of primary tissue significantly influences the success rate of organoid cultures. The most challenging aspects of PDO culture include optimizing culture conditions, limited tumor tissue sampling, uncertainty in medium composition, lack of a TME, as well as contamination risks and ethical controversies during the culture process [24–29]. These challenges necessitate continuous exploration and improvement to increase the success rate and broaden the application potential of BC PDO cultures. The following sections will discuss advancements and prospective applications of breast organoids by subtype, focusing on their role in understanding various BC types and developing therapeutic strategies (Figure 1B).

### Organoid and luminal BC

**Figure 2.**
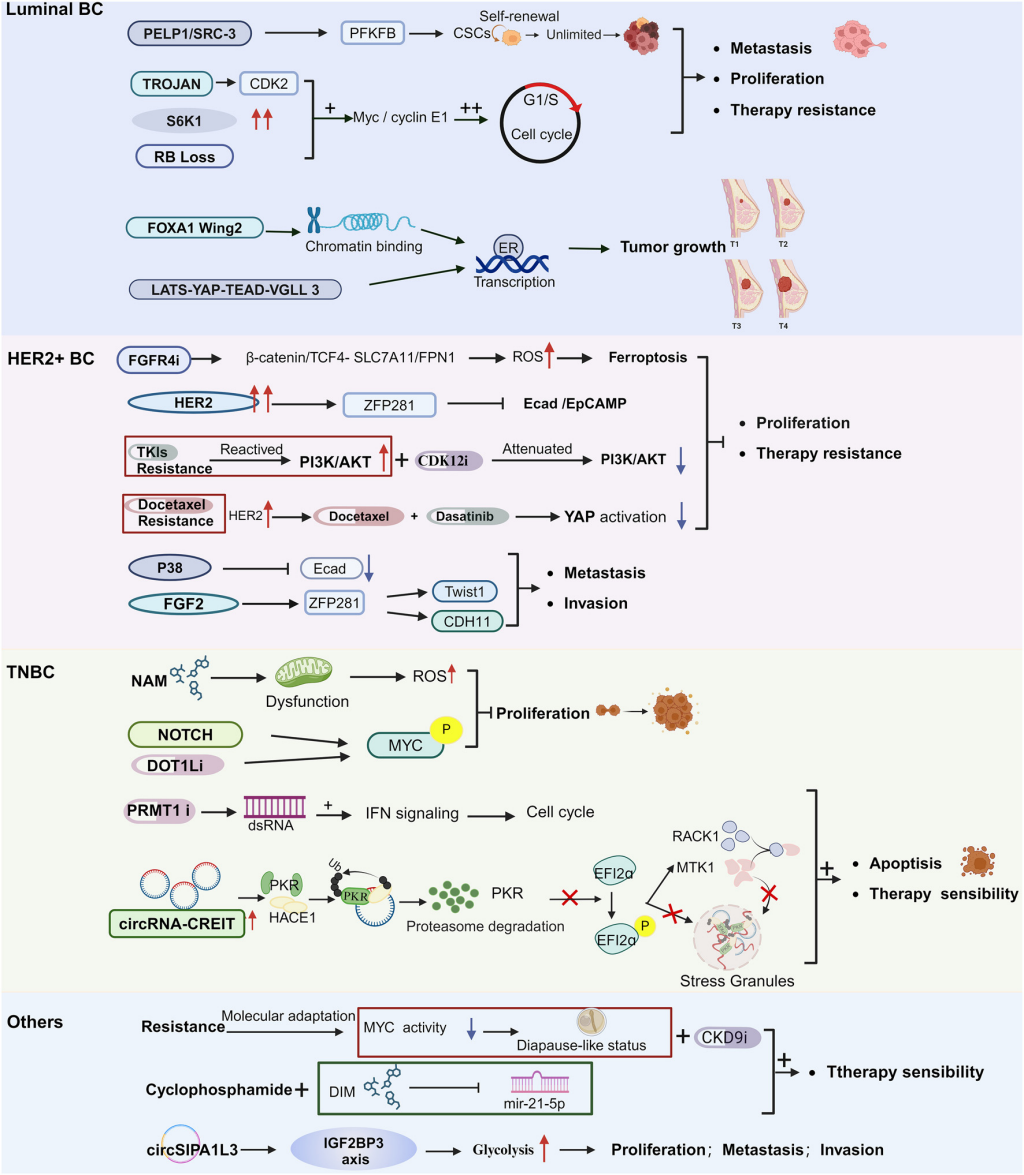
**Overview of the results of research based on the use of various subtypes of BC organs, including basic mechanisms and therapeutic studies.** The figure shows important genes or signaling pathways related to the development and drug resistance of various subtypes of breast discovered through organoids, and suggests available therapeutic targets and the mechanism of action of new drugs. BC: Breast cancer.

Luminal BC, accounting for roughly 70% of all BC cases, poses considerable challenges due to its high likelihood of distant metastasis, recurrence, and resistance to endocrine therapy, all of which contribute to elevated patient mortality [30]. To better understand the molecular factors driving these challenges, as well as the mechanisms of drug resistance and preclinical treatment responses linked to Luminal BC, researchers are increasingly utilizing organoids as a powerful investigative tool (Figure 2, Table 1).

**Table 1:** Effective novel therapeutic drugs, compounds, and therapeutic combinations based on organoid discovery

	**Targeted drug/Compound**	**Combined therapy**
Luminal BC	• PFKFB inhibitor • PELP1 / SRC-3 complex inhibitor (SI-2/5MPN) • LATS inhibitor (e.g., VT02956) • HDAC inhibitor (e.g., Entinostat) • Birinapant (representative drug of apoptosis inhibitor protein inhibitors)	• Tam + SI-2/5MPN • BCL2 selective inhibitor (ABT-199) + Fulvestrant + CDK4/6 inhibitor • RB-deficient: Aliserti + MK1775 • CD4/6 inhibitor + mTOR inhibitor
HER2+ BC	• FGFR4 inhibitors • CDK12 inhibitors • ZFP281 transcription factor	• FGFR4 inhibitor + anti-HER2 therapy • Docetaxel + Avastat in/Dasatinib • CDK12 inhibitor + lapatinib
TNBC	• In situ dendritic cell vaccine (HELA-Exos) • Nicotinamide (NAM) • MS023 • DOT1L inhibitor EPZ - 5676	• ICB + metabolites • GPX4 inhibitor + immunotherapy
Others	• FGFR4 inhibitor (BLU9931) • CDK9 inhibitor • Resveratrol (RES) • 3-3’-Diindolylme thane (DIM) • Oxypalmatine (OPT)	• DIM + methotrexate/cycl ophosphamide

The table presents a range of current organoid-based results in breast cancer drug therapy, which can inform the development of new drugs and combinations of new drugs, thereby facilitating the provision of personalised treatment options for patients. BC: Breast cancer; TNBC: Triple negative breast cancer.

#### Basic mechanisms of luminal BC

Organoids have emerged as indispensable tools for studying the molecular factors governing the occurrence, progression, and therapeutic resistance of Luminal BC. Studies by Dekkers et al. successfully generated Luminal BC organoids using breast basal progenitor cells and luminal progenitor (LP) cells. Interestingly, attempts to target a combination of three TS genes (TP53, PTEN, RB1, and NF1) using the CRISPR-Cas9 system failed to produce organoids. However, reintroducing these genes resulted in the successful construction of Luminal BC organoids that were responsive to endocrine therapy and chemotherapy [17]. This highlights the potential of combining the organoid model with gene editing for precision therapy. In a related study, lentivirus-transduced mutations of TP53 and PTEN in normal BC organoids were transplanted into mice, resulting in the development of Luminal-type BC. This approach identified key driver genes for this BC subtype [22]. Similarly, Bhatia et al. [31] observed PIK3CA mutations in most Luminal PDOs while establishing an organoid database. Gene editing, deletion, and reintroduction of PIK3CA confirmed its role as a genetic driver in Luminal BC organoid formation, emphasizing the heterogeneity within this BC subtype and the importance of individualized treatment approaches. These findings suggest that research on organoids to identify BC oncogenes remains limited. Future studies should integrate gene editing technologies with BC organoids to explore additional driver genes and therapeutic targets, which could facilitate early diagnosis and timely intervention.Truong et al. used Luminal BC PDOs to reveal that PELP1/SRC-3 and PFKFB kinase collaboratively drive tumor stem cell proliferation and therapeutic drug resistance. By leveraging 2D cell lines, mouse tumor cells, and PDO models, they conducted immunohistochemical analysis, showing that a PFKFB inhibitor combined with ER-targeted therapy effectively blocked tumor sphere formation in advanced BC models [17]. This demonstrates the utility of organoid models in analyzing the therapeutic sensitivity of various inhibitor combinations and selecting optimal therapies.

Jin et al. employed PDO and PDX models to demonstrate that inhibiting TROJAN enhances the efficacy of CDK4/6 inhibitors by blocking S-phase entry mediated by CDK2 activation. Their findings supported the use of anti-TROJAN antisense oligonucleotides (ASOs) in combination with CDK4/6 inhibitors to significantly inhibit tumor progression, underscoring the potential of organoids in therapeutic research [32]. Aruabarrena et al. revealed that the FOXA1 Wing2 mutation in Luminal BC sustains estrogen response and reduces the therapeutic efficacy of aromatase inhibitors (AIs). Luminal BC organoids derived from genetically engineered mice were instrumental in delineating the mutation’s impact on chromatin binding and ER transcription, providing insights into therapeutic challenges associated with this BC subtype [13, 33]. The above studies demonstrate the potential of PDOs in therapeutic research, complementing 2D cell culture models and PDX.

#### Therapy of luminal BC

Addressing the challenge of endocrine therapy resistance in Luminal BC, organoids play a pivotal role in investigating drug resistance mechanisms, conducting drug screening, exploring therapeutic targets, and guiding clinical decisions. Walsh’s team pioneered the use of PDOs to guide the selection of the most effective drug combinations, laying the foundation for targeted combination therapy research and clinical trials for Luminal BC. Subsequently, Chew et al. extended the use of Luminal BC PDOs by evaluating the combination of BCL2 inhibitors, fluvastatin, and CDK4/6 inhibitors [34, 35]. Their results demonstrated the superior efficacy of triplet combination therapy over monotherapy, laying the groundwork for investigating targeted combination therapies in Luminal BC. In drug sensitivity experiments using PDX and PDO models, Chew et al. [36] identified FGFR4 as a potential target for treating advanced endocrine-resistant Luminal BC, paving the way for clinical drug screening. Using CRISPR/Cas9 technology, Ma et al. discovered that LATS deletion inhibits ESR1 transcription through the LATS-YAP-TEAD-VGLL3 axis (Hippo-YAP pathway) in Luminal BC PDOs lacking the Hippo pathway kinase LATS. This inhibition suppresses cancer cell and PDO growth [37]. In another study, Li Dan’s team identified significant S6K1 overexpression in drug-resistant PDOs treated with pazopanib. Further research revealed that mTOR inhibitors can reverse this resistance and synergize with CDK4/6 inhibitors, offering a promising therapeutic strategy [38]. Kumarasamy et al. uncovered that RB deletion in various Luminal BC models results in selective drug resistance. Using PDOs, they found that a combination of Alisertib and MK1775 induced apoptosis in an RB deletion environment, suggesting a potential therapeutic approach for RB-deficient Luminal BC [39]. Building upon these findings, Navarro-Yepes team investigated the mechanisms of Palbociclib resistance (PR) and Abemaciclib resistance (AR) using PDX and PDX-derived organoids. They discovered that patients with acquired PR resistance can benefit from combined AR [40]. This highlights the utility of PDOs in elucidating resistance mechanisms and guiding therapeutic strategies. Numerous studies confirm that PDOs provide a valuable platform for drug testing. Constructing target-specific PDOs by adding relevant cytokines and pathway inhibitors in culture not only validates potential therapeutic targets but also provides an effective strategy for preclinical drug testing.

### Organoid and HER2+ BC

HER2+ BC presents significant challenges, particularly due to the development of acquired resistance to anti-HER2 therapies. In this context, organoids have emerged as essential tools for unraveling the mechanisms of drug interactions in HER2+ BC, addressing drug resistance, and facilitating the development of more effective targeted treatments (Figure 2, Table 1).

#### Metastasis mechanisms of HER2+ BC

Metastasis remains a major driver of BC-related mortality. Pioneering research by Harper and colleagues in 2016 revealed the role of P38 in HER2+ BC, demonstrating its ability to prevent the downregulation of E-cadherin. This process fosters the development of early disseminated cancer cells (eDCCs) and acts as a barrier to early metastasis. Subsequent work by Nobre et al. utilized PDOs and *in vivo* mouse models to explore the function of the ZFP281 transcription factor. Their findings highlighted its role in inducing long-term dormancy in eDCCs, thereby regulating cancer cell dissemination and distant metastasis [41, 42]. These studies emphasize the critical importance of organoids in advancing our understanding of drug resistance mechanisms in HER2+ BC and in unraveling the complexities of metastatic progression.

#### Treatment of HER2+ BC

Using HER2+ PDOs, Li Hui’s team demonstrated that inhibiting CDK12 attenuates PI3K/AKT signaling, thereby enhancing the therapeutic efficacy of lapatinib through downregulation of the PI3K pathway [15]. Similarly, Zou team found that inhibiting FGFR4 phosphorylation—associated with m6A hypomethylation—regulates β-catenin/TCF4 signal transduction, effectively overcoming HER2 resistance. In HER2+ BC cases with drug resistance, combined FGFR4 and anti-HER2 inhibition exhibited a synergistic effect [43]. Sachs and Shudan’s teams utilized PDOs as platforms for drug screening to evaluate the sensitivity of HER2+ BC patients to HER2-targeted therapies and assess their responses to neoadjuvant chemotherapy. Additionally, Duarte et al. employed BRCA1-mutant HER2+ BC PDOs to assess sensitivity to PARP inhibitors [13, 21, 27]. Rowe and Daley identified heterogeneous PDOs resistant to docetaxel treatment, which led to the discovery of a combination therapy involving Avastin or Dasatinib [11]. This combination restored sensitivity to docetaxel by inhibiting mechanical signaling and YAP activation. Notably, these findings were consistent with clinical patient responses, highlighting the potential of PDOs to predict neoadjuvant chemotherapy outcomes and serve as valuable platforms for drug testing. These studies emphasize the predictive power of PDOs in determining neoadjuvant chemotherapy responses, their utility in drug screening, and their potential to guide individualized treatments. While case reports have shown PDO models benefiting patients with lung and liver cancer [44, 45], there are currently no documented cases of their use in BC clinical treatment. This may be attributed to factors, such as BC staging, grading, patient age, and marital or childbearing history, which complicate modeling and follow-up efforts. Nevertheless, ongoing research continues to explore the application of PDOs in BC drug screening and treatment optimization.

### Organoid and triple-negative BC (TNBC)

#### Basic mechanism of TNBC

To address the significant challenge of BC heterogeneity, particularly in TNBC, Bhatia et al. [31] established a diverse biological sample bank of BC PDOs, primarily derived from TNBC cases. Through comprehensive analyses—including genome, transcriptome, and cell morphology studies—they identified key carcinogenic mutations, such as PIK3CA, TP53, and KMT2C, shedding light on the pathogenesis of TNBC. The team also uncovered the overactivation of NOTCH and MYC signaling pathways in tumor LP-like cells compared to their normal counterparts. Using PDO models, they validated the targeted effects of NOTCH overactivation on TNBC proliferation and invasion [31]. Comparative analysis between normal breast organoids and TNBC PDOs provided valuable insights into dysregulated genes and pathways in cancer, advancing the understanding of tumor initiation and progression. Moreover, gene editing of PDOs allowed for the validation of candidate-specific targets and invasion-related pathways, significantly enhancing our knowledge of the driving and invasive mechanisms of TNBC (Figure 2).

#### Treatment of TNBC

Exploring treatment strategies for TNBC using PDOs has provided valuable insights into anti-tumor mechanisms and novel therapeutic approaches. Wu et al. [46] investigated the anti-tumor mechanisms of type I PRMT inhibitors using TNBC PDOs and demonstrated that these inhibitors induce double-stranded RNA production, triggering an interferon response. Similarly, Kurani et al. utilized TNBC PDOs derived from PDXs to confirm that DOT1L inhibitors impede tumor cell proliferation and division by reducing c-Myc expression and inhibiting ALDH1 stem cell activity. These findings present a promising therapeutic strategy for TNBC [47]. PDOs have also proven effective for identifying combinatory drug strategies. Conway et al. [48] used TNBC PDOs to show that simultaneous inhibition of the glucocorticoid receptor (GR) and signal transducer and activator of transcription 3 (STAT3) synergistically inhibits TNBC cell growth. Their work identified effective drug combinations, such as SH4-54 and mifepristone, which could guide future treatment development. Jung et al. explored the effects of NAM treatment on TNBC PDOs, revealing mitochondrial dysfunction and the activation of reactive oxygen species (ROS) through the reverse electron pathway. Their findings clarified NAM’s therapeutic potential and suggested that PDOs could help optimize NAM dosage for individualized treatment [49]. Furthermore, Wang et al. [50] investigated candidate circular RNAs (circRNAs) in TNBC using both animal models and PDOs. CircRNAs, previously studied in rectal, gastric, and BCs [51–53], hold promise for identifying patient-specific therapeutic targets. Notably, circRNA-CREIT emerged as a potential biomarker for BC diagnosis and prognosis. Targeting circRNA-CREIT and stress granule formation was shown to enhance TNBC chemosensitivity. Continued use of PDOs for identifying and validating patient-specific targets may open new avenues for TNBC treatment. In the realm of immunotherapy, TNBC PDOs have also proven invaluable. Professor Jiang Yizhou’s team demonstrated the susceptibility of LAR-type TNBC cells to GPX4 inhibitors, suggesting that combining GPX4 inhibitors with immunotherapy could represent a novel strategy for this subtype [54]. Meanwhile, Huang et al. [55] developed an in situ dendritic cell vaccine (HELA Exos) using TNBC organoids, which promoted the activation of dendritic cells and tumor-responsive CD8+ T cells, yielding significant anti-tumor activity. Additionally, the Shelkey team created an immune-enhanced tumor organoid (iTO) model to study factors influencing immune checkpoint blockade (ICB) responses [56]. This model assessed the effects of bacterial metabolites on ICB efficacy, incorporating analyses of immune cell number and activity, cytotoxicity, and genotype changes. Their findings highlight the model’s potential for guiding immunotherapy strategies. Although organoid-based studies are not yet widely employed directly in clinical settings, they offer promising opportunities to advance TNBC treatments, guide personalized therapies, and improve patient outcomes. As research continues, we can expect an increasing number of reports that leverage PDOs to enhance clinical approaches.

### Others

Organoids have emerged as an innovative and appealing model for disease research, offering significant advancements in BC studies. Despite some discrepancies with the human microenvironment, organoids serve as valuable preclinical models, providing a novel platform for drug discovery, investigation, and the development of treatment strategies for BC. As organoid technology matures, its applications extend beyond subtype-specific BC research, encompassing broad-spectrum anti-BC drugs, non-specific metastasis mechanisms, and other areas of study (Figure 2, Table 1). Organoids have proven invaluable for uncovering drug resistance and metastasis mechanisms across various BC types. For example, McLachlan et al. discovered that gap junction proteins (Cx26, Cx43) regulate endothelial angiogenesis, inhibiting tumor progression and metastasis through non-gap junction intercellular communication (GJIC)-dependent mechanisms. This was demonstrated using MB-231 cell-derived organoids [57]. Similarly, Dhimolea et al. [58] employed PDOs to investigate the MYClow diapause state induced by chemotherapy, a phenomenon linked to chemotherapy resistance. Using PDOs resistant to cytotoxic chemotherapy drugs (TP organoids), they tested the efficacy of candidate drugs and identified CDK9 inhibitors as effective in reversing the diapause-like state caused by Myc activity inhibition. This, in turn, enhanced chemotherapy efficacy. These findings highlight the potential of organoids for large-scale drug screening and provide critical insights into reversing chemotherapy resistance. Further research is needed to explore how the MYClow diapause state may promote metastasis and/or chemotherapy resistance, with organoids serving as a valuable tool for such studies. Patients with advanced BC often face challenges from multiple drug resistance, necessitating the development of low-toxicity, high-efficacy anti-tumor drugs. Resveratrol (RES), a natural polyphenol compound found in grapes and peanuts, has demonstrated efficacy and low toxicity in treating PDOs derived from various subtypes of advanced BC. Ye et al. [59] discovered that RES selectively targets both STAT3-dependent and STAT3-independent BC types, underscoring its potential for personalized treatment based on patients’ STAT3 signaling status. Nikulin et al. cultivated organoid cells from metastatic tissues of three BC subtypes to test the therapeutic effects of 3-3’-diindolylmethane (DIM). DIM inhibited the expression of mir-21-5p, enhanced chemosensitivity in PDOs, and exhibited synergistic effects with commonly used chemotherapy drugs. These findings suggest DIM’s potential role in preclinical and clinical studies as part of standard treatment regimens [14]. Using BC cell lines, mouse models, and BC PDOs, Liang et al. investigated the effects of circSIPA1L3 on BC cell proliferation, migration, invasion, and stemness. Their study revealed the oncogenic effects of circSIPA1L3, mediated through glucose metabolism pathways. Additionally, Daan Smits, Peter D. Haughton, and Thijs Koorman explored the role and mechanisms of YAP in cancer cell invasion using 3D culture systems, immunofluorescence imaging, single-cell mRNA sequencing, and pharmacological intervention experiments. While organoid models provide invaluable insights, ethical concerns remain regarding the removal of original tumor samples from patients, presenting a significant clinical dilemma [18, 19]. However, the integration of cellular models, animal models, and organoid models has created a powerful experimental platform for studying the biological properties and invasion mechanisms of BC tumor cells. Combining PDX with PDOs is also emerging as a promising pathway. For instance, Guillen et al. [20] utilized paired PDX and PDXO models for drug studies, mitigating some ethical controversies associated with PDXO use. To address these challenges, increased public awareness and education about the benefits of organoid research may foster patient cooperation, ensuring adherence to a human-centered approach. Government and hospital organizations could play a key role in promoting these efforts, paving the way for broader acceptance and application of organoid-based technologies in clinical and research settings.

## Summary and outlook

Organoids have emerged as invaluable tools for studying the development of various BC subtypes, elucidating drug resistance mechanisms, exploring therapeutic targets, developing novel drugs and combinations, and investigating personalized treatment strategies. This review provides a detailed analysis of organoid applications in BC research, categorized by subtypes. While organoids have become a standard platform for drug sensitivity screening in precision tumor treatment, several challenges persist. A substantial number of samples and repeated experiments are often required for mechanism research and preclinical prediction of treatment responses, which poses practical limitations. Additionally, heterogeneity in gene expression and artificial differentiation during experimental processes makes it difficult to ensure consistent genomics and tissue morphology across organoid batches, presenting obstacles for certain studies. Addressing issues of homogenization and reproducibility is critical to unlocking the full potential of organoids in broader mechanism-based research. Furthermore, current BC organoids lack systemic integration, with insufficient incorporation of vascular tissue, immune cells, and nervous system elements to adequately mimic growth and metastatic processes. This limitation hinders their application in studying immunotherapeutic and anti-vascular drugs. Ethical concerns also arise regarding the use of organoids in research and clinical contexts, particularly when anonymizing patient data is unfeasible [60−63]. Without a solution, organoids cannot be used effectively for long-term generational studies or clinical applications. To build a larger and more diverse organoid sample pool, it is essential to establish a robust informed consent model that balances institutional and privacy protections. Resolving this ethical dilemma may require coordinated efforts, including public awareness campaigns and science communication initiatives by governments and healthcare organizations, to increase patient cooperation and uphold a human-centered approach. Looking ahead, the efficient application of organoids in precision medicine, basic research on mechanisms, and clinical translation depends on continuous efforts to expand organoid sources and optimize cultivation conditions. Future research is expected to focus on using organoids to investigate cell fate plasticity, understand the molecular basis of aging and regeneration, advance organ transplantation and regenerative medicine, and more. These advancements are likely to drive the development of novel therapeutic approaches, biological or immunotherapeutic technologies, and innovative methods for diagnosing and treating BC.

According to recent reports, microfluidic chip technology can partially address the absence of a vascular system in organoids. Microfluidic chips have been utilized to assist in establishing lung organoids, stomach organoids, and others [64, 65]. While the combination of microfluidic technology and BC research is still under exploration and not yet widely reported, it has attracted significant attention from research teams. This suggests that microfluidic chips can serve as *in vitro* culture platforms for organoids, providing valuable opportunities to study cell behavior in heterogeneous populations and complex microenvironments. Such technology may emerge as a powerful tool for advancing studies on human PDOs, disease modeling, drug screening, and regenerative medicine, potentially opening new possibilities for organ transplantation. In conclusion, advanced gene-editing techniques, co-culture of adult stem cells, single-cell RNA sequencing, microfluidics, and the inclusion of specific cytokines could help establish more clinically relevant PDOs. This progress has the potential to greatly expand the applications of organoids, particularly in experimental research and drug development. The ongoing advancements in organoid technology hold great promise for transforming the landscape of BC research, reshaping diagnostic approaches, and revolutionizing treatments.
